# Risks associated with continuation of potentially inappropriate antihypertensive medications in older adults receiving hemodialysis

**DOI:** 10.1186/s12882-021-02438-3

**Published:** 2021-06-19

**Authors:** Rasheeda K. Hall, Sarah Morton, Jonathan Wilson, Patti L. Ephraim, L. Ebony Boulware, Wendy L. St. Peter, Cathleen Colón-Emeric, Jane Pendergast, Julia J. Scialla

**Affiliations:** 1 Department of Medicine, Duke University School of Medicine, Box DUMC 2747, 2424 Erwin Road Suite 605, Durham, NC 27710 USA; 2 Durham Veterans Affairs Medical Center, Durham, NC USA; 3 Biostatistics and Bioinformatics, Duke University School of Medicine, Durham, NC USA; 4 Department of Epidemiology, Johns Hopkins University Bloomberg School of Public Health, Baltimore, MD USA; 5 Department of Pharmaceutical Care and Health Systems, University of Minnesota College of Pharmacy, Minneapolis, MN USA; 6 Departments of Medicine and Public Health Sciences, University of Virginia School of Medicine, Charlottesville, VA USA

## Abstract

**Background and objectives:**

After dialysis initiation, older adults may experience orthostatic or post-dialysis hypotension. Some orthostasis-causing antihypertensives (i.e., central alpha agonists and alpha blockers), are considered potentially inappropriate medications (PIMs) for older adults because they carry more risk than benefit. We sought to (1) describe antihypertensive PIM prescribing patterns before and after dialysis initiation and (2) ascertain the potential risk of adverse outcomes when these medications are continued after dialysis initiation.

**Design, setting, participants, and measurements:**

Using United States Renal Data System data, we evaluated monthly prevalence of antihypertensive PIM claims in the period before and after dialysis initiation among older adults aged ≥66 years initiating in-center hemodialysis in the US between 2013 and 2014. Patients with an antihypertensive PIM prescription at hemodialysis initiation and who survived for 120 days were classified as ‘continuers’ or ‘discontinuers’ based on presence or absence of a refill within the 120 days after initiation. We compared rates of hospitalization and risk of death across these groups from day 121 through 24 months after dialysis initiation.

**Results:**

Our study included 30,760 total patients, of whom 5981 (19%) patients had an antihypertensive PIM claim at dialysis initiation and survived ≥120 days. Most [65% (*n* = 3920)] were continuers. Those who continued (versus discontinued) were more likely to be black race (26% versus 21%), have dual Medicare-Medicaid coverage (31% versus 27%), have more medications on average (12 versus 9) and have no functional limitations (84% versus 80%). Continuers experienced fewer all-cause hospitalizations and deaths, but neither were statistically significant after adjustment (Hospitalization: RR 0.93, 95% CI 0.86, 1.00; Death: HR 0.89, 95% CI: 0.78–1.02).

**Conclusions:**

Nearly one in five older adults had an antihypertensive PIM at dialysis initiation. Among those who survived ≥120 days, continuation of an antihypertensive PIM was not associated with increased risk of all-cause hospitalization or mortality.

## Introduction

Although older adults comprise more than half of the US incident hemodialysis population [1], more than 50% die and/or spend an average of a month hospitalized in the first year of dialysis [2, 3]. Factors that are associated with these adverse outcomes among older adults initiating dialysis are often non-modifiable (e.g., advanced age, comorbidities) [4]; however, more evidence is needed to identify modifiable risk factors, especially for those who survive the first few months of dialysis. One modifiable factor known to be associated with hospitalization and mortality in older adults is use of potentially inappropriate medications (PIMs). According to the American Geriatrics Society (AGS) Beers Criteria, PIMs are defined as medications that generally confer greater risk than benefit in older adults [5]. Prior studies suggest deprescribing (i.e. stopping and/or lowering the dose) PIMs could improve polypharmacy and related hospitalizations in older adults receiving dialysis [6, 7]. Antihypertensive PIMs listed in the AGS Beers Criteria, specifically central alpha agonists and alpha blockers, could be medications to target for deprescribing in patients receiving dialysis because there are alternative antihypertensives [5, 8]. It is unknown if this guidance should be applied to prescribing decisions for older adults initiating dialysis who have both a greater risk of both cardiovascular events and medication side effects than the general population.

Evidence suggests that antihypertensive PIMs may be risky for older adults. Both central alpha agonists and alpha blockers frequently cause orthostatic hypotension which can be exacerbated if relative volume depletion is present after dialysis. Central alpha agonists can also cause sedation [9]. Both hypotension and sedation increase the risk of falls and fall-related complications, including hospitalizations or death [10]. For an older adult starting dialysis, hypotension during dialysis may further increase the risk of falls and injury [11]. Conversely, older adults on dialysis also have an increased risk of cardiovascular events and mortality. There is also a U-shaped association of blood pressure and mortality in dialysis patients [12]. Therefore, the relative risks versus benefits of using antihypertensive PIMs in older adults receiving dialysis is an important unanswered question.

Because hemodialysis can impact blood pressure, dialysis initiation often presents an important transition in clinical care when antihypertensives are reviewed or changed (e.g., discontinuation of diuretics). However, some antihypertensives are not changed at all, perhaps due to clinical inertia or lack of evidence to inform clinical decision-making [13]. Evidence to inform the use of antihypertensive PIMs is therefore needed. The aims of this study were to (1) describe prescribing patterns of antihypertensive PIMs before and after dialysis initiation, and (2) quantify the potential risk of hospitalization and death associated with continuation of these medications after dialysis initiation.

## Methods

### Study design

We conducted a retrospective cohort study to describe national trends in prescribing antihypertensive PIMs around dialysis initiation. In a sub-cohort of individuals, we further estimated risks associated with continuing or discontinuing antihypertensive PIMs after dialysis initiation. We used the United States Renal Data System (USRDS) data including Medicare claims (Parts A, B, and D) to establish both the full and sub-cohorts and ascertain clinical characteristics, clinical events, and prescriptions. Medicare, the US federal health insurance program, has three main components that can be used for epidemiologic studies in dialysis patients: (1) Part A covers hospitalizations, skilled nursing, and hospice care, (2) Part B, an optional program, covers outpatient care, physician services, and medical supplies, and (3) Part D, an optional prescription program, covers prescription costs [14]. This study was approved by the Duke University Institutional Review Board (Pro00100199) and the USRDS. Given the retrospective nature of this study and the deidentified data, informed consent was waived for this study by the Duke University Institutional Review Board (Pro00100199). All methods were performed in accordance with relevant regulations and guidelines.

### Study population

Full Study Population. From the USRDS, we identified a full cohort of adults aged ≥66 years who (a) initiated in-center hemodialysis between January 1, 2013 and Dec 31, 2014, and (b) had Medicare Part A and B as primary payer and Part D coverage for at least 1 year prior to dialysis initiation. Medicare insurance coverage is generally allowed after the 65th birthday or for younger patients who qualify after diagnosis with end-stage kidney disease requiring kidney replacement therapy or meet other criteria for insurance benefits. Therefore, we selected age ≥66 years for cohort entry to ensure that prospective cohort members, older adults new to dialysis, were eligible to receive Medicare for at least 1 year which increases likelihood of available clinical claims for analyses. We excluded patients who received hospice care during the 6 months prior to dialysis initiation, resulting in a full cohort of 30,760 individuals. The full study cohort was used to summarize prevalence trends in prescribing central alpha agonists and alpha blockers.

Sub-cohort for Health Outcomes. Using the full cohort, we identified a sub-cohort who survived at least 120 days after dialysis initiation and had at least one Part D prescription claim for a central alpha agonist and/or alpha blocker at the time of dialysis initiation. The sub-cohort was used in outcome analyses of hospitalization rates and mortality risk. For both prevalence and outcome analyses, patients were right-censored for death, hospice initiation, modality switch (i.e., kidney transplantation, peritoneal dialysis, or home hemodialysis), or the end of follow up, 24 months after dialysis initiation.

### Identification of PIMs

Antihypertensive PIMs were identified from the Part D claims data using national drug codes (NDC) that correspond to hierarchical generic product identifier codes (GPI) obtained from Wolters Kluwer’s Medi-Span® Electronic Drug File (MED-FILE) Version 2 (Indianapolis, IN, USA). GPI codes starting with 362010 and 362020 represent central alpha agonists and alpha blockers, respectively. Corresponding with these GPI codes, the central alpha agonists included clonidine, methyldopa, and guanfacine; the alpha blockers included terazosin, prazosin, and doxazosin.

### Defining PIM exposure

To assess PIM exposure in the full cohort (prevalence analyses), we identified each Medicare Part D prescription claim with NDCs for either a central alpha agonist or alpha blocker and its associated ‘days supplied’ beginning 6 months prior to dialysis initiation (day −180) and ending 24 months afterwards (day 720). Because we assumed patients may not use their home supply during a hospitalization, patients were considered to be using the medication for the days supplied expressed over outpatient days and with a 15 day grace period to allow for late refills. For the purpose of monthly point prevalence analyses, PIM exposure was defined as having days’ supply on the 15th day of each 30-day period (‘month’). Because PIM exposure was defined by Part D claims, patients were removed from the active cohort (for prevalence analyses) during months that they experienced gaps in Medicare Part A, B, or D enrollment, hospitalization, or days spent in a skilled nursing facility (SNF). Patients returned to the active cohort when Medicare coverage resumed and after hospitalization or SNF stays.

To assess PIM exposure in the sub-cohort (outcome analyses), we first established this sub-cohort as individuals with evidence of an antihypertensive PIM at dialysis initiation defined by having a Part D prescription claim with NDCs for either a central alpha agonist or alpha blocker that had a days’ supply that overlapped with the date of dialysis initiation. We examined for overlap by examining the days’ supply of a prescription claim and the date of dialysis initiation. A patient was considered to have an overlapping prescription if on the date of dialysis initiation, their supply of central alpha agonist and/or alpha blocker ≥1 or they were in a 15 day grace period for their central alpha agonist and/or alpha blocker. We observed for ≥1 additional antihypertensive PIM prescription claim in the 120 days after dialysis initiation. We assumed that providers reconcile and adjust medications around the time of dialysis initiation and thus used an intention to treat principle to capture the intention of treating providers. We classified those with and without an additional claim in the 120 days after dialysis initiation as continuers and discontinuers, respectively. We did not further update medication status over time because subsequent decisions may reflect tolerance or failure of the initial treatment strategy which could induce bias. We selected the 120 day period to best capture discontinuation even among those receiving a 90 day supply of the medication. We also selected this 120 day period to minimize survivor bias and confounding related to both high mortality rates after dialysis initiation and features of early death after dialysis initiation, such as insufficient pre-dialysis nephrology care or congestive heart failure [15, 16].

### Hospitalization and mortality outcomes

**Fig. 1:**
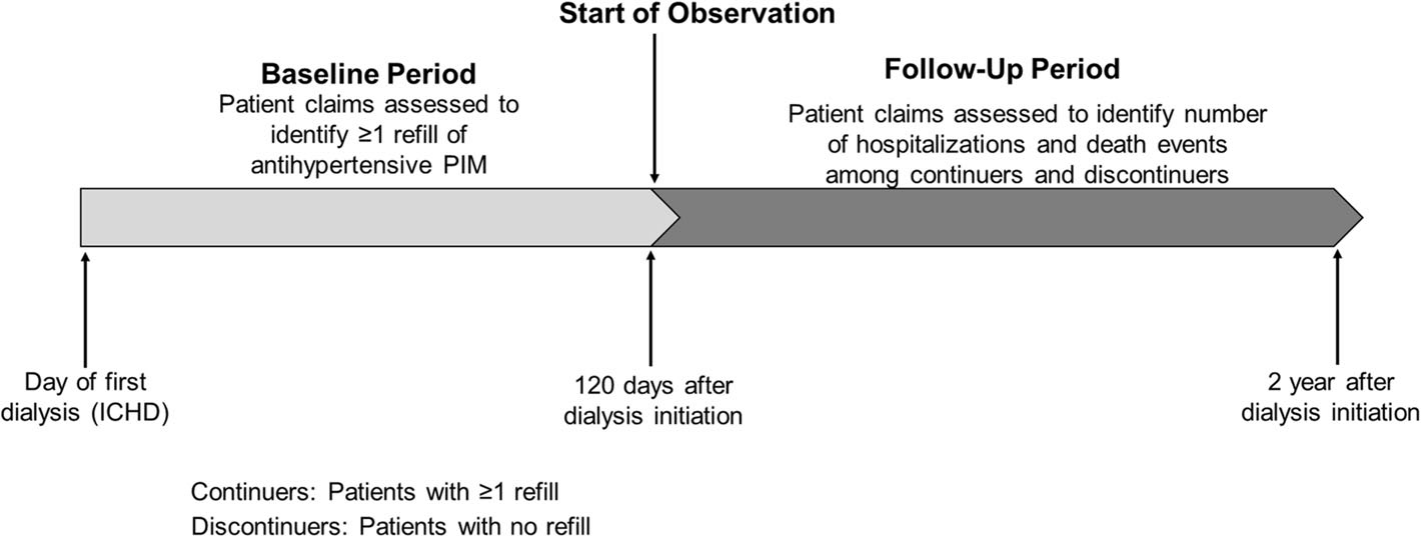
Study Diagram for Longitudinal Data Analyses. A sub-cohort of patients with ≥1 prescription for an antihypertensive potentially inappropriate medications (PIMs) overlapping with day of first dialysis were included in analyses with observation from 120 days to 2 years after dialysis initiation if not censored

In the sub-cohort of patients classified as continuers or discontinuers of antihypertensive PIMs after dialysis initiation, we examined hospitalization and mortality (Fig. 1). All-cause hospitalizations were identified through Medicare Part A inpatient claims. Death dates were ascertained from the CMS Form 2746 in the USRDS Core SAFs. The observation time period for hospitalization rates and mortality started on the 121st day after dialysis initiation and ended 24 months after dialysis initiation, or when follow-up was permanently censored. Interval censoring for hospitalization was used to adjust time at risk for a new hospitalization by removing hospitalized days from time at risk. Raw hospitalization rates were then defined as the number of hospital admissions divided by the number of person-years at risk.

### Covariates

Information on sociodemographic, clinical, and health system characteristics at the time of dialysis initiation were ascertained from the USRDS Core Dataset, including the Center for Medicare and Medicaid Services (CMS) Medical Evidence Form (2728), Patients Standard Analytic File (SAF), Payer History SAF, Facility SAF, and ESRD (End-stage renal disease) Medicare payment data. Socio-demographics included age, gender, race/ethnicity, dual-eligibility status (both Medicare and Medicaid coverage) as indicated in USRDS Patient and Payer History SAFs. Clinical characteristics included the Liu comorbidity index score, a comorbidity index developed based on a cohort of dialysis patients with Medicare insurance coverage computed from evidence of 11 comorbid conditions (atherosclerotic heart disease, congestive heart failure, cerebrovascular accident/transient ischemic attack, peripheral vascular disease, other cardiac, chronic obstructive pulmonary disease, gastrointestinal bleeding, liver disease, dysrhythmia, cancer, and diabetes) based on inpatient and outpatient claims in the year prior to dialysis initiation [17], the number of hospitalizations in 12 months prior to dialysis initiation from Medicare Part A inpatient claims, and the number of unique medications prescribed in the 120 days after dialysis initiation (excluding topical medications, eye drops, diabetic supplies and vaccinations) from Part D claims [18]. Additional clinical characteristics obtained from CMS 2728 included presence/absence of diabetes, ESRD cause, an indicator of functional limitation (defined as presence of at least one of the following three variables: inability to ambulate, inability to transfer, or need of assistance with daily activities), and pre-dialysis nephrology care. Health system characteristics included the facility’s for-profit status, a facility characteristic that has previously been associated with adverse outcomes [19], and geographic region (as determined by ESRD network) as indicated on the Facility SAF.

### Statistical analyses

For prevalence analyses in the full cohort, monthly point prevalence of central alpha agonist and alpha blocker prescriptions in 30-day intervals from 6 months prior to 24 months after dialysis initiation were computed using the number of cohort members with prescriptions claims and number of active (non-censored) cohort members at each timepoint. We report the point prevalence graphically by type of PIM—individually, in combination, and overall.

For outcome analyses in the sub-cohort, we performed summary statistics describing characteristics of the sub-cohort as counts (%) for categorical variables and as either means (standard deviations (SD)) or medians (interquartile ranges (IQR)) for continuous variables. These summary statistics were presented for continuers, discontinuers, and combined. The average hospitalization rates for the two study groups (continuers/discontinuers) were compared using general linear model assuming a negative binomial distribution for observed counts and log link. The negative binomial distribution is very similar to the more commonly used Poisson distribution, but is preferred when the variance in the counts exceeds the mean. Cox proportional hazard models were used to assess the relationship between continuing a central alpha agonist or alpha blocker and mortality. Assumptions of proportional hazards were evaluated visually with Kaplan-Meier plots. Initial models were adjusted for covariates as described above, including interaction between antihypertensive PIM continuation and functional limitation. We performed post-hoc analyses to test for interaction between antihypertensive PIM continuation and age group in two categorizations: (1) five age groups: 66–70, 71–75, 76–80, 80–85 and ≥86 years and (2) dichotomized age groups: ages 66–75 and age ≥76 years. Model results presented do not include non-significant interaction terms, or cases missing one or more covariate values (3%). Final models included adjustment for all sociodemographics, clinical characteristics, and health system characteristics described above. All statistical analyses were performed using SAS/STAT(c) 15.1 (SAS Institute, Cary NC) and statistical tests were conducted at the 0.05 level.

## Results

### Prevalence of antihypertensive PIM prescriptions before and after dialysis initiation

**Fig. 2:**
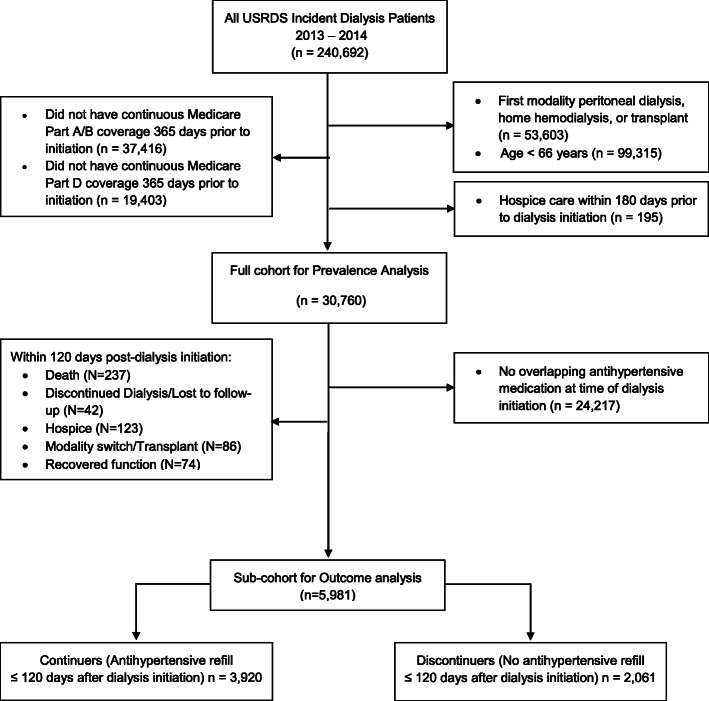
Consort Diagram. The full cohort (*n* = 30,760) was used to describe trends in prescriptions for antihypertensive potentially inappropriate medications (PIMs). A sub-cohort (n = 5981) with evidence of antihypertensive PIM at dialysis initiation were included in statistical analyses

**Fig. 3:**
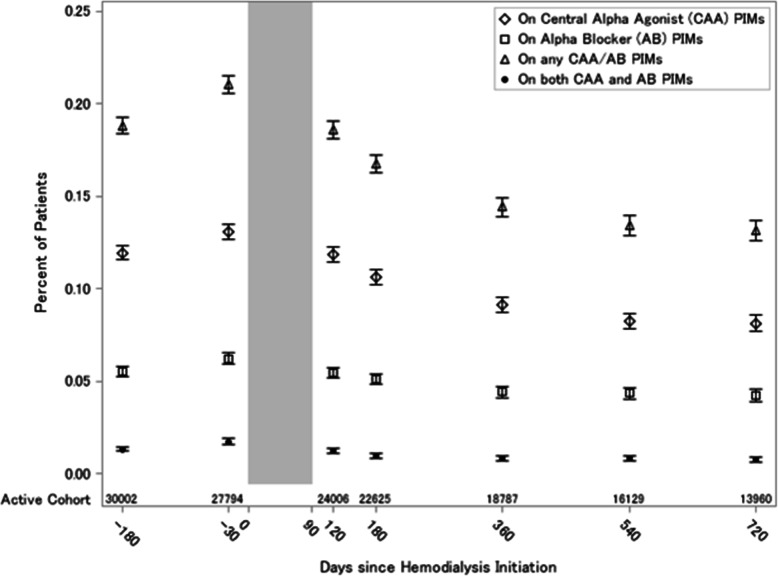
Proportion of Patients with Prescriptions for Antihypertensive Potentially Inappropriate Medications (PIMs) Prior to and After Dialysis Initiation among the Full Cohort. The figure shows proportion of patients with an antihypertensive potentially inappropriate medication (PIM) claim among those who were eligible at the given time point from 6 months before to 24 months after dialysis initiation. The gray vertical bar indicates months with significant interval censoring for hospitalizations and skilled nursing facility admissions leading to less accurate estimation of PIM exposure. The number of active cohort members at each time point is indicated at the bottom of the figure. Prevalence estimates represent serial point prevalence and may reflect changes in the cohort membership over time rather than intentional discontinuation of the medication

Of 240,692 incident dialysis patients in 2013 and 2014, we identified 30,760 patients who met eligibility criteria for our prevalence analyses (Fig. 2). We examined monthly prevalence of antihypertensive PIMs at select months. At 6 months prior to dialysis initiation, 19% (*n* = 5645) of 30,002 active cohort members had an antihypertensive PIM prescription: 12% (*n* = 3582) had a central alpha agonist (predominantly clonidine), 6% (*n* = 1661) had an alpha blocker, and 1% (*n* = 402) had both. At 1 month prior to dialysis initiation, 21% (*n* = 5850) of 27,794 active cohort members had an antihypertensive PIM prescription with a similar distribution across types (Fig. 3). This relative “increase” may be related to cohort effects, in which the number of patients in the total analytic population changes due to permanent censoring, hospitalizations and SNF admissions. After dialysis initiation, point prevalence falls stabilizing around 13–19% (Fig. 3).

### Characteristics of the outcomes cohort

Among the full cohort of 30,760, 19% (*n* = 5981) had antihypertensive PIM claim overlapping with dialysis initiation and survived the first 120 days of dialysis (Fig. 2). In this sub-cohort used for outcome analyses, the majority (65%, *n* = 3920) were continuers of antihypertensive PIMs, based on having a refill for an antihypertensive PIM in the first 120 days of dialysis (Table 1). Compared to those who discontinued an antihypertensive PIM, those who continued were more likely to be younger [75 (6) versus 76 (7) years], have Non-Hispanic black race/ethnicity (26% versus 21%), have dual Medicare-Medicaid insurance coverage (31% versus 27%), have more prescriptions medications [12 (5) versus 9 (5)], and have no functional limitations (84% versus 80%; Table 1).

**Table 1:** Cohort Demographics and Covariates by Presence of Antihypertensive PIM Refill after Dialysis Initiation in the Sub-Cohort

	Total (n = 5981)	Continued (*n* = 3920)	Discontinued (*n* = 2061)	*p*-value
**Age at first ESRD service**				<0.001
Mean (SD)	76 (6)	75 (6)	76 (7)	
**Female**	3077 (51%)	2011 (51%)	1066 (52%)	0.75
**Race/Ethnicity**				<0.001
Non-Hispanic white	3450 (58%)	2164 (55%)	1286 (62%)	
Non-Hispanic black	1469 (25%)	1031 (26%)	438 (21%)	
Hispanic	703 (12%)	488 (12%)	215 (10%)	
Other	354 (6%)	237 (6%)	117 (6%)	
**Medicare/Medicaid Dual Eligible status**	1795 (30%)	1233 (31%)	562 (27%)	0.001
**Comorbidity Index**				<0.001
Mean (SD)	8.0 (4.1)	7.6 (4.1)	8.8 (4.0)	
Median (IQR)	8.0 (5.0, 11.0)	8.0 (4.0, 11.0)	9.0 (6.0, 12.0)	
**Diabetes**	4560 (76%)	3001 (77%)	1559 (75%)	0.43
**Medication count**				<0.001
Mean (SD)	11 (5)	12 (5)	9 (5)	
Median (IQR)	11 (8, 14)	12 (9, 15)	9 (6, 12)	
**Functional limitations**	1038 (17%)	622 (16%)	416 (20%)	<0.001
**Nursing home residence**	577 (10%)	292 (7%)	285 (14%)	<0.001
**Pre-dialysis nephrology care**^a^	4194 (70%)	2807 (72%)	1387 (67%)	0.005
**Pre-dialysis hospitalizations**^b^				<0.001
Mean (SD)	1.6 (1.9)	1.5 (1.9)	1.7 (1.9)	
Median (IQR)	1.0 (0.0, 2.0)	1.0 (0.0, 2.0)	1.0 (0.0, 2.0)	
**For-Profit Facility**	5171 (86%)	3407 (87%)	1764 (86%)	0.16
**Facility Geographic Region**				0.16
Northeast	1007 (17%)	657 (17%)	350 (17%)	
South	2442 (41%)	1594 (41%)	848 (41%)	
Midwest	1341 (22%)	870 (22%)	471 (23%)	
West	1045 (17%)	713 (18%)	332 (16%)	
Unknown	146 (2%)	86 (2%)	60 (3%)	
**ESRD Cause**				0.04
Diabetes	2928 (49%)	1949 (50%)	749 (47%)	
Hypertension	2305 (38%)	1507 (38%)	798 (39%)	
Glomerulonephritis	246 (4%)	165 (4%)	81 (4%)	
Other	502 (8%)	299 (8%)	203 (10%)	

Data presented as N(%) or as indicated by row

*ESRD* end-stage renal (kidney) disease

^a^Pre-dialysis nephrology care defined as presence of care by nephrologist prior to dialysis initiation irrespective of length of care

^b^Pre-dialysis hospitalization defined as number of hospitalizations in 12 months preceding dialysis initiation

### Risk of hospitalizations and mortality

Patients were observed over a median follow-up of 609 days. In unadjusted analyses, those who continued (versus discontinued) had lower risk of all-cause hospitalization [RR 0.87 (0.80, 0.93)] and death [HR 0.93 (0.86, 1.00)] (Table 2). However, those who continued (versus discontinued) antihypertensive PIMs after dialysis initiation had only a marginally lower risk of all-cause hospitalization after adjustment for sociodemographics, ESRD cause, presence of diabetes, nursing home residence, pre-dialysis nephrology care, medication count, comorbidity index, functional limitation, and facility region and for-profit status [adjusted RR 0.93, 95% CI (0.86, 1.00)]. Those who continued (versus discontinued) no longer had a lower risk of death after adjustment for the same covariates (adjusted HR 0.89, 95% CI: 0.78–1.02). We did not find statistical evidence that functional limitation modified the association between continuing an antihypertensive PIMs and either adverse outcome (*p*-interaction 0.45 for hospitalization and 0.19 for mortality). Similarly, age group did not modify the association between continuing an antihypertensive PIM and either adverse outcome (*p*-interaction 0.56 for hospitalization and 0.23 for mortality for 5 year age group models, *p*-interaction 0.49 for hospitalization and 0.72 for mortality for dichotomized age group models).

**Table 2:** Risk for Hospitalization and Mortality Associated with Antihypertensive PIM Use

	Discontinued Medication	Continued Medication
**Hospitalization**		
Total in Analysis	2060	3920
Total person time at risk (person years)	2525.4	5161.5
No. of Events	4203	7939
Rate-per-person-years	1.66 (1.61, 1.71)	1.54 (1.44, 1.64)
Unadjusted RR	Reference	0.87 (0.80, 0.93)
Adjusted RR^a^	Reference	0.93 (0.86, 1.00)
**Mortality**		
Total in Analysis	2061	3920
No. of Events (%)	431 (21%)	679 (17%)
Unadjusted HR	Reference	0.77 (0.69, 0.87)
Adjusted HR^a^	Reference	0.89 (0.78, 1.02)

Abbreviations: *PIM* potentially inappropriate medications, *CI* confidence interval, *HR* hazard ratio, *RR* rate ratio

^a^Models were adjusted for demographics, dual Medicare and Medicaid eligibility, comorbidity index, diabetes, ESRD cause, hospitalization count in prior 12 months, pre-dialysis nephrology care, facility for-profit status/region, nursing home residence, and functional limitation. Due to its recurrent nature, negative binomial regression was used to model hospitalization yielding rate ratios. Cox proportional hazards regression was used to model mortality yielding hazard ratios

## Discussion

In a nationally representative cohort of older adults initiating hemodialysis, we found antihypertensive PIM claims were present in 19% prior to dialysis initiation. In a sub-cohort of older adults who had an active antihypertensive PIM at the time of dialysis initiation and survived 120 days after dialysis initiation, the majority (65%) continued an antihypertensive PIM after dialysis initiation. While those who continued had fewer comorbidities and were less likely to have functional impairment than those who discontinued, adjusted analyses did not demonstrate clear evidence of an association of continuing PIMs with risk of hospitalization or mortality. Although not definitive, these findings suggest that central alpha agonists and alpha blockers are not PIMs to prioritize for deprescribing in older adults receiving hemodialysis.

Our findings on antihypertensive PIM use are consistent with prior evidence, demonstrating approximately 1 in 5 older adults had antihypertensive PIMs before dialysis initiation, and this proportion declined after dialysis initiation [20]. There is minimal evidence on the efficacy of central alpha agonists and alpha blockers for hypertension in advanced chronic kidney disease and dialysis patients yet these medications are likely added for patients having difficult to control hypertension while taking multiple antihypertensive medications [21]. Over time after dialysis initiation, there was a lower point prevalence of both central alpha agonists and alpha blockers. This finding could be explained by: (1) cohort effects (i.e., death events, modality switch, or hospice) or (2) improved blood pressure control and serum potassium levels after dialysis initiation allowing use of only other antihypertensives, such as beta blockers and renin angiotensin system medications, which are more commonly prescribed in dialysis patients and for which clinical trials support their use [22, 23]. Our outcome analyses do not suggest antihypertensive PIMs increase risk of hospitalization or mortality, but there may still be adverse outcomes associated with prescribing PIMs in older adults receiving dialysis. It remains that antihypertensive PIMs may increase risk of falls, cognitive impairment, or patient reported outcomes, such as fatigue and dizziness, which also should be considered in prescribing decisions. There are additional AGS Beers Criteria PIMs, such as anticholinergic antidepressants, that may individually (or in combination with antihypertensive PIMs) associate with hospitalization and mortality [24]. Therefore, the pursuit to identify specific PIMs that are modifiable risk factors for poor prognosis in older adults receiving dialysis remains needed. Because physical well-being is highly valued by dialysis patients, additional studies are needed to determine an association between antihypertensive PIMs and risk of frailty and/or functional impairment. Such evidence would substantiate clinical trials evaluating the efficacy of deprescribing on clinical outcomes in this vulnerable population.

The findings from our outcome analyses contrast with existing guidance and prior studies suggesting harm associated with antihypertensive PIM use in the general older population. The AGS Beers Criteria strongly advises that central alpha agonists and alpha blockers be avoided as routine therapy for hypertension based on low or moderate quality of evidence, or evidence derived mostly from observational studies or case-control studies [5]. Such prior studies have shown central alpha agonists and alpha blockers to be associated with hospitalizations in the general older adult population [25, 26]. Our findings may differ from these prior studies as we examined outcomes among individuals who were all taking these agents at some point while some discontinued them. It is possible that characteristics of individuals who take these medications may differ from those who do not take them. For example, those who take these medications may have more difficult to control hypertension just prior to initiating hemodialysis. So far as our study included a distinct cohort from those prior studies—older adults receiving hemodialysis who carry high risk for intermittent hypotension, cardiovascular events secondary to uncontrolled hypertension, and mortality. Our findings may also differ from analyses taking other approaches because we chose to minimize survivor bias (given the high mortality after dialysis initiation) by analyzing data from individuals who survived 120 days of dialysis [16]. While this approach does not focus on adverse outcomes during a highly vulnerable period, it does allow the findings to inform deprescribing priorities for the broader prevalent population of older adults receiving dialysis. Still, we have to acknowledge confounding by selective prescribing as we identified continuers had less functional impairment than discontinuers [27]. Providers may discontinue antihypertensive PIMs among patients who appear more vulnerable (i.e., hemodynamic instability). Overall, this study’s findings imply that additional pharmacoepidemiologic studies that address this confounding (e.g., propensity score or by restricting the cohort to patients without hemodynamic instability) may yield more clarity on the risk attributable to antihypertensive PIMs [28].

This study’s strengths lie in the use of a nationally representative cohort of older adults receiving dialysis and our rigorous approach to medication ascertainment (use of Medi-Span® to obtain comprehensive list of national drug codes) and adjustment variables (use of pre-ESRD claims and development of functional limitation variable) to support causal inference. However, our study has limitations. First, we examined Medicare Part D claims to ascertain medication possession counts of antihypertensive PIMs, however prescriptions dispensed do not fully reflect actual use of these medications. Second, we did not utilize a matched cohort design to minimize treatment selection bias. Still, this bias was reduced in part by our selection of meaningful covariates. Third, our approach with an intention-to-treat framework did not analyze any later discontinuation or re-initiation of a PIM. As a result, our comparison groups may not be absolutely distinct as continuers and discontinuers. Nonetheless, this intent-to-treat approach should result in more conservative estimates compared to an as-treated analysis. Fourth, while we assessed hospitalizations because they represent significant complications from falls, falls cannot accurately be captured directly from claims data [29]. As a result, we could not assess difference in fall risk between continuers and discontinuers. Last, our models relied on claims data and therefore did not capture unmeasured clinical confounders, including concurrent antihypertensives, blood pressure control, intradialytic hypotension, and other time-varying clinical measures that may impact both decisions regarding continuation of antihypertensives and our outcomes, hospitalizations and mortality. Future studies could strengthen or refute our findings by including these unmeasured confounders with dialysis unit electronic medical record data and controlling for selection bias.

In conclusion, we found that continuation of central alpha agonists and/or alpha blockers after dialysis initiation in clinically selected patients was not associated with increased risk of hospitalizations or death among older adults who survived at least 120 days of hemodialysis. While our results suggest that providers could continue individualized use of these agents in clinical decision-making for blood pressure management, further studies are needed to better capture the potential adverse effects of these medications and other PIMs on important clinical outcomes for the older dialysis population.

## Data Availability

The datasets generated and analyzed during the current study have been supplied by the United States Renal Data System (USRDS) but restrictions apply to the availability of these data, which were used under data use agreement for the current study, and so are not publicly available.
